# Potential chemopreventive, anticancer and anti-inflammatory properties of a refined artocarpin-rich wood extract of *Artocarpus heterophyllus* Lam.

**DOI:** 10.1038/s41598-021-86040-5

**Published:** 2021-03-25

**Authors:** Isaac J. Morrison, Jianan Zhang, Jingwen Lin, JeAnn E. Murray, Roy Porter, Moses K. Langat, Nicholas J. Sadgrove, James Barker, Guodong Zhang, Rupika Delgoda

**Affiliations:** 1 Natural Products Institute, The University of West Indies, Mona, Jamaica West Indies; 2 Department of Basic Medical Sciences, Faculty of Medical Sciences, University of the West Indies, Mona, Jamaica, Jamaica West Indies; 3 Department of Food Science, University of Massachusetts, Amherst, MA USA; 4 Department of Chemistry, The University of West Indies, Mona, Jamaica West Indies; 5 Jodrell Laboratory, Department of Natural Capital and Plant Health, Royal Botanic Gardens, Kew, Richmond, TW9 3DS UK; 6 School of Life Sciences, Pharmacy and Chemistry, Kingston University, Penrhyn Road, Kingston-upon-Thames, Surrey UK

**Keywords:** Biochemistry, Cancer, Plant sciences, Diseases, Molecular medicine

## Abstract

Colorectal cancer (CRC) represents the third leading cause of death among cancer patients below the age of 50, necessitating improved treatment and prevention initiatives. A crude methanol extract from the wood pulp of *Artocarpus heterophyllus* was found to be the most bioactive among multiple others, and an enriched extract containing 84% (*w*/*v*) artocarpin (determined by HPLC–MS–DAD) was prepared. The enriched extract irreversibly inhibited the activity of human cytochrome P450 CYP2C9, an enzyme previously shown to be overexpressed in CRC models. In vitro evaluations on heterologously expressed microsomes, revealed irreversible inhibitory kinetics with an IC_50_ value of 0.46 µg/mL. Time- and concentration-dependent cytotoxicity was observed on human cancerous HCT116 cells with an IC_50_ value of 4.23 mg/L in 72 h. We then employed the azoxymethane (AOM)/dextran sodium sulfate (DSS) colitis-induced model in C57BL/6 mice, which revealed that the enriched extract suppressed tumor multiplicity, reduced the protein expression of proliferating cell nuclear antigen, and attenuated the gene expression of proinflammatory cytokines (*Il-6* and *Ifn-γ*) and protumorigenic markers (*Pcna*, *Axin2*, *Vegf*, and *Myc)*. The extract significantly (*p* = 0.03) attenuated (threefold) the gene expression of murine *Cyp2c37*, an enzyme homologous to the human CYP2C9 enzyme. These promising chemopreventive, cytotoxic, anticancer and anti-inflammatory responses, combined with an absence of toxicity, validate further evaluation of *A. heterophyllus* extract as a therapeutic agent.

## Introduction

Colorectal cancer (CRC) ranks as the third most commonly diagnosed cancer in the US^1^ and in Jamaica^2^, and it is the second leading cause of cancer-related deaths in both sexes in the US^1^. While CRC is affected by modifiable factors such as environment, physical activity, Quetelet's index, diet, cigarette smoking, and alcohol consumption^3^, it is of noteworthy concern that it ranks as the third leading cause of death among young cancer patients aged 20–49 in the US^4^. Approximately 20% of patients diagnosed with colorectal cancer in Jamaica between 2008 and 2012 were younger than 50 years old^5^. A recent study on a murine model showed that moving away from a Western diet even after carcinogenesis may reduce tumor burden^6^, demonstrating that incorporating dietary compounds containing anticancer, antioxidant, and anti-inflammatory properties may have added benefits in treating and preventing colorectal cancer. Given that nature has inspired significant solutions to cancer therapy in the past four decades^7^ and the demonstrated reliance and confidence on ethnomedicines by cancer patients^8^, it is imperative that nature’s sources be fully evaluated as novel therapeutic options for the treatment and prevention of CRC.

Cytochrome P450 (CYP) enzymes are ubiquitous enzymes considered highly important in the metabolism of pharmaceutical drugs^9,10^, as well as in the activation of environmental carcinogens^11^. The upregulation of CYP enzymes in cancer tissue in comparison to surrounding tissue implies their vital role in carcinogenesis, as well as in drug resistance^12^. The overexpression of CYP2C enzymes in murine induced CRC models, as well as in human CRC cell lines^13^ and human CRC tissue samples^14^, in comparison to their normal counterparts, suggests a role for these enzymes in pathogenesis, due most likely to the increased amount of carcinogenic metabolites derived from their activities. Indeed, elevated levels of epoxygenated fatty acids in the plasma of mice with induced CRC provide evidence for such biomarker upregulation^13^. These findings lend support to the search for targeted CYP2C inhibitors that could mitigate enzyme activities, thus functioning as chemopreventors^15^, in addition to the search for prodrugs that rely on activation from elevated metabolic enzyme activity^16^.

The *Artocarpus* genus, comprising nutrient-rich fruits with ethnomedicinal uses in Southeast Asia, Africa, Central America, and the Caribbean, demonstrates numerous potential health benefits. Isolated phytochemicals including artocarpin, isoartocarpin, cyloartocarpin, artocarpetin, and norartocarpetin were found to have anti-inflammatory, anticancer, chemopreventive, and antioxidant properties, as well as inhibit the cellular production of melanin^17^. A previous study^18^ revealed the targeted impact of isolated artocarpin on the phosphoinositide 3 -kinase/Akt pathway, suggesting a chemopreventive role. Yet, there is a dearth of information on its impact on cytochrome P450 2C enzymes, previously found to have elevated levels in CRC models^14^. Furthermore, a more rigorous in vivo study examining the inflammatory response is still needed, particularly through the evaluation of a bioactive extract that can display a high safety profile. Hence, we employed a multitarget study design to better understand the potential colon cancer-reducing effects of an *A. heterophyllus* extract. Following numerous in vitro evaluations using heterologously expressed human CYP enzymes, as well as human cancer cell lines, we used the AOM/DSS model, a chemically induced colitis-associated cancer (CAC) mouse model employing azoxymethane (AOM) and dextran sulfate sodium (DSS) carcinogens, which mimics a form of inflammatory colorectal cancer in humans, to assess the impact of the *A. heterophyllus* extract on colon tumor development^19,20^.

## Results

### Identification and standardization of bioactive *A. heterophyllus* extract

**Figure 1:**
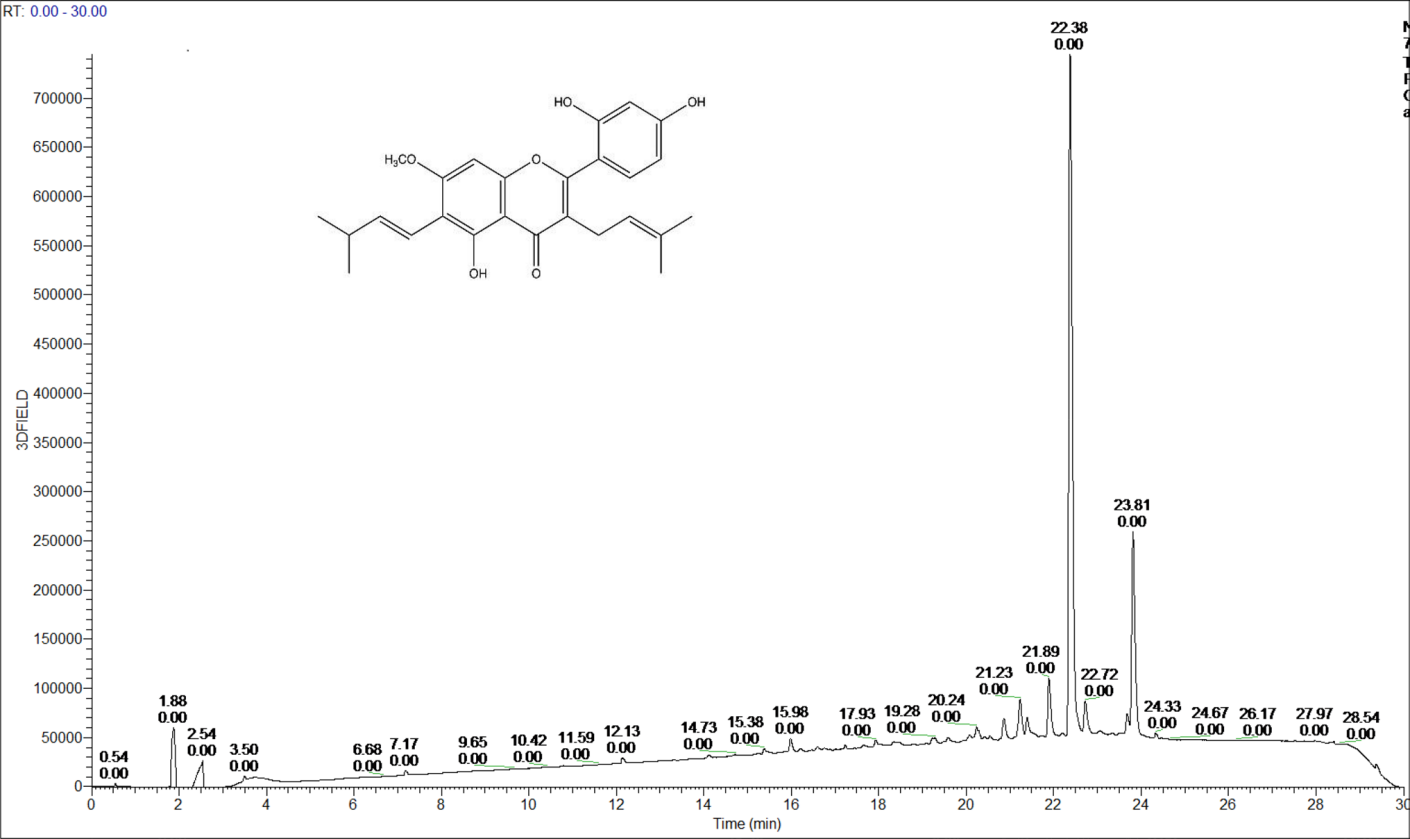
HPLC–MS chromatogram of the *A. heterophyllus* Lam. extract containing artocarpin (inset).

Eight crude extracts were prepared from various parts of the *A. heterophyllus* Lam. plant with *n*-hexane and methanol. Then, after an initial screen for cytotoxicity potential, the crude methanol extract from the wood was identified as the most potent. This crude extract was fractionated using column chromatography, which led to 14 semi-purified fractions. Through the application of a cytotoxic assay using a panel of three cell lines (Supplementary Table S1), those with potent bioactivity was identified. Accordingly, an enriched extract was prepared using solvent extraction. The final enriched extract (simply referred to as the *A. heterophyllus* extract from here on) was quantified as containing 84% (*w*/*v*) artocarpin using LC–MS (Fig. 1). This was confirmed by NMR analysis and a standard curve using the UV absorption of pure artocarpin.

**Figure 2:**
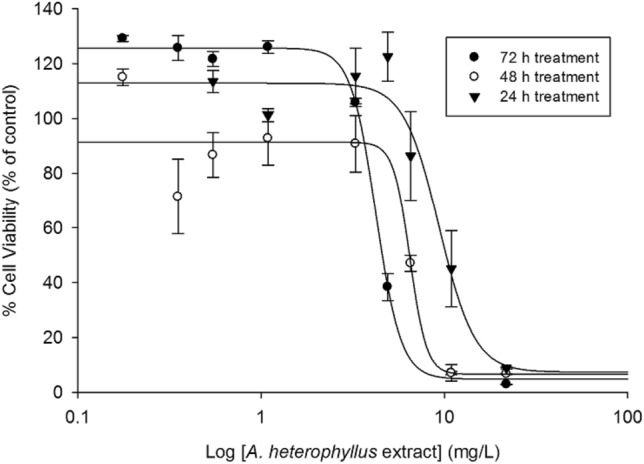
Cytotoxicity of the *A. heterophyllus* extract toward human colon adenocarcinoma HCT116 cells. The cells were treated with the *A. heterophyllus* extract for 24 h, 48 h, and 72 h, and the cell viability, as a percentage relative to the solvent (ethanol)-treated control, was measured using the MTT assay as detailed in the methods. Data are presented as the mean ± SEM of three independent experiments.

The prepared *A. heterophyllus* extract was then evaluated for cytotoxicity using the aggressive, human CRC cell line, HCT116. Cell viability decreased in a concentration- and time-dependent manner in the presence of the *A. heterophyllus* extract. After 24 h of treatment with the *A. heterophyllus* extract, an IC_50_ value of 9.38 ± 1.26 mg/L was obtained, while, after 48 h and 72 h of exposure, the IC_50_ decreased by 31% and 55% to 6.48 ± 0.63 and 4.23 ± 0.08 mg/L, respectively (Fig. 2).

### Impact of *A. heterophyllus* extract on CYP2C enzyme activity in vitro and in vivo

**Figure 3:**
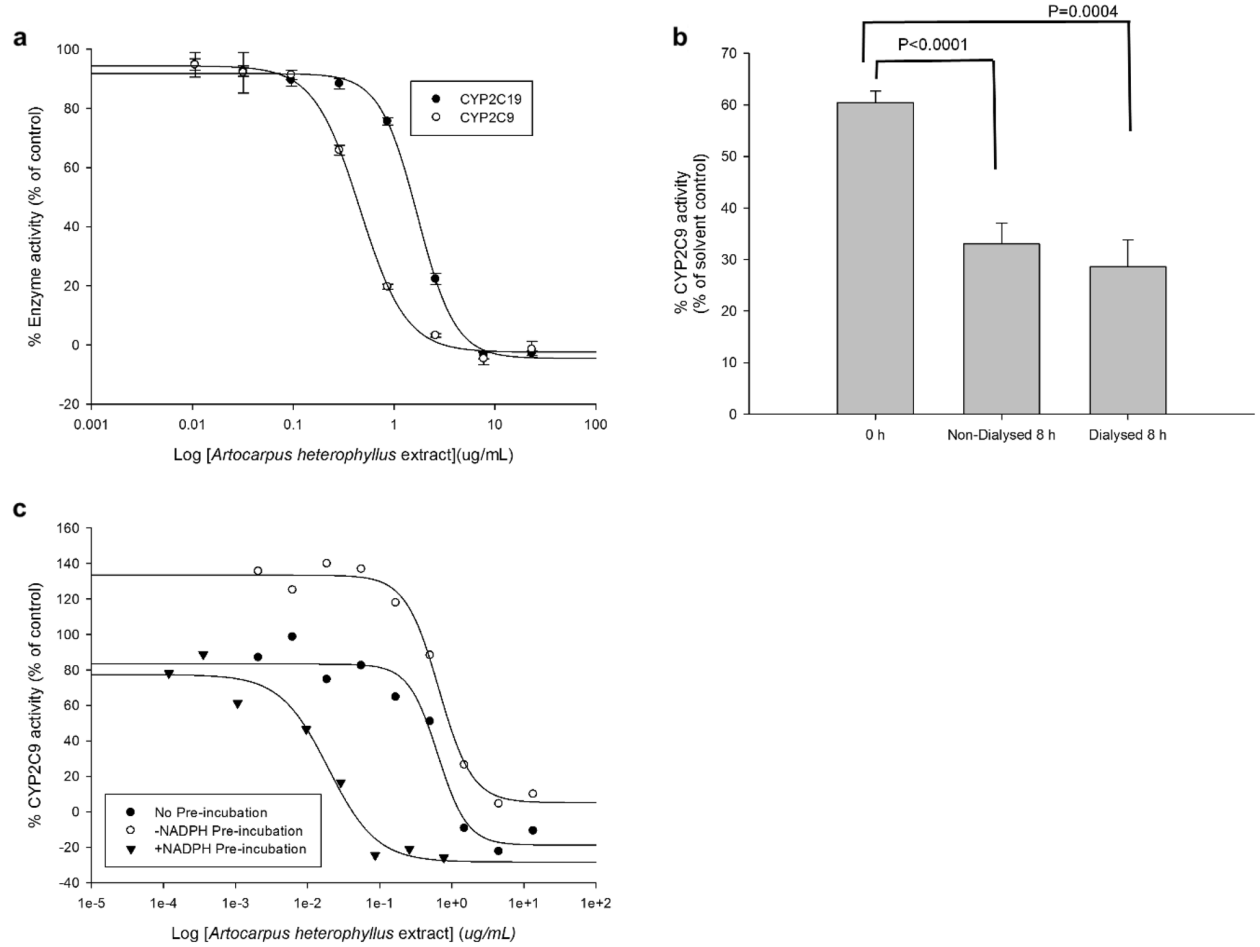
Inhibitory effects of the *A. heterophyllus* extract on human CYP2C9-mediated 7-methoxy-4-trifluoromethylcoumarin demethylase (80 µM) and CYP2C19-catalyzed 3-cyano-7-ethoxycoumarin deethylase (25 µM) in vitro activities. (**a**) *A. heterophyllus* extract concentration-dependent inhibition of CYP2C9 and CYP2C19 activities. Control activities performed with methanol (mean ± SEM) for CYP2C9 and 2C19 were 5.84 × 10^−4^ ± 3.56 × 10^−5^ and 8.01 × 10^−3^ ± 2.83 × 10^−4^ µM metabolite formed per min/pmol of CYP, respectively. (**b**) Effect of dialysis on the inactivation of human recombinant CYP2C9 activity by the *A. heterophyllus* extract. For (**a**) and (**b**), each data point is the mean percentage of control enzyme activity for three independent experiments. A *p* value ≤ 0.05 was considered statistically significant. (**c**) Effect of preincubation in the presence or absence of NADPH on the inhibition of human recombinant CYP2C9 activity by the *A. heterophyllus* extract.

Given the putative role of CYP2C enzymes in the pathogenesis of CRC in humans and mice ^13^, we evaluated the inhibitory potential of the *A. heterophyllus* extract on the activities of human CYP2C9 and 2C19 enzymes. Potent, concentration-dependent inhibition was observed against the activity of heterologously expressed human CYP2C9 with an IC_50_ of 0.46 ± 0.02 µg/mL, while moderate inhibition was observed against the activity of CYP2C19, with an IC_50_ value of 1.71 ± 0.09 µg/mL (Fig. 3a). Further characterization of the inhibition revealed irreversible kinetics against CYP2C9 with no return in activity, even after 8 h of dialysis following exposure to the extract (Fig. 3b). A shift in IC_50_ of 33 units (Fig. 3c) following a 30-min preincubation is suggestive of time-dependent kinetics, although further timepoint evaluations are needed to validate such assertions.

**Figure 4:**
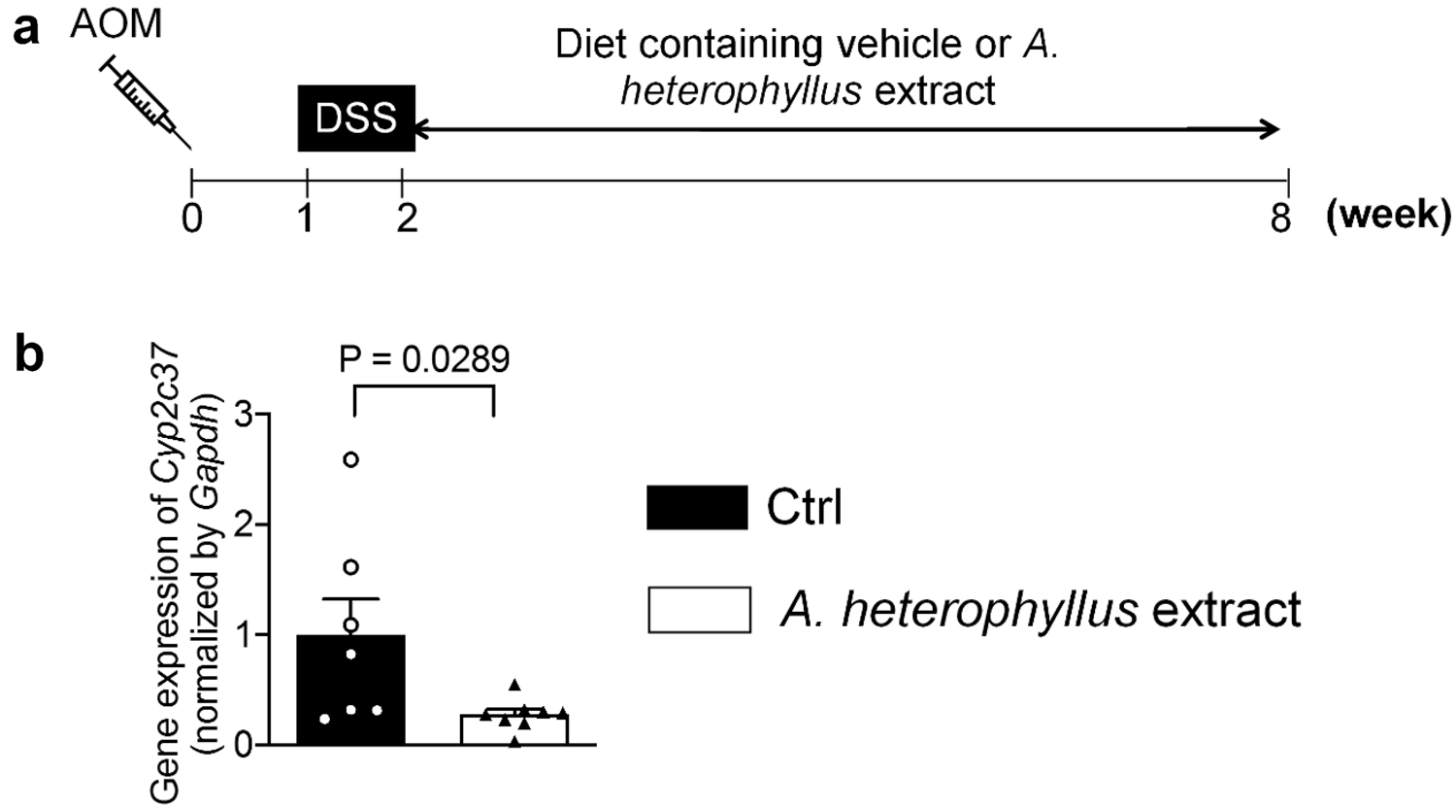
qRT-PCR evaluation of the *Cyp2c37* gene expression in vivo*.* (**a**) Scheme of animal experiment. (**b**) qRT-PCR analysis of *Cyp2c37* gene expression in colon tissues. The results are expressed as the mean ± SEM, *n* = 11 mice in Ctrl group and *n* = 10 mice in the *A. heterophyllus* group. Ctrl, vehicle control.

The above in vitro evaluations pointed to an effective and potent impact of the wood extract of *A. heterophyllus* Lam. on markers of chemoprevention and cytotoxicity, motivating its further evaluation using an in vivo model. We, therefore, implemented the AOM/DSS colitis-induced cancer mouse model to gain additional insight into in-vivo efficacy (see scheme of animal experiment in Fig. 4a).

The mice with AOM/DSS colitis-induced cancer who were administered the *A. heterophyllus* extract displayed a marked threefold reduction (*p* = 0.02) in the expression of *Cyp2c37* gene throughout the colon (Fig. 4b), gauged by qRT-PCR, in comparison to the vehicle control diet group. This gene was selected due to its high homology with the human *CYP2C9* gene (as *cyp2c9* is not found in mice). The observed reduction in *Cyp2c37* gene expression, along with an inhibitory effect toward the enzyme activity of the human protein product (CYP2C9), suggests a potential role for the *A. heterophyllus* extract in chemoprevention via the CYP2C pathway.

### Impact of *A. heterophyllus* extract on AOM/DSS-induced colon tumorigenesis

The effect of the *A. heterophyllus* extract containing artocarpin was tested on AOM/DSS-induced colon tumorigenesis in mice. The mutagenic activity of AOM requires metabolic activation by CYP2E1^21^. Thus, the *A. heterophyllus* extract was included in the diet, after allowing the colonic mutagen AOM enough time to be activated by CYP2E1, coupled with DSS-induced colonic inflammation, thereby mimicking colitis-associated tumorigenesis (see scheme of animal experiment in Fig. 4a).

**Figure 5:**
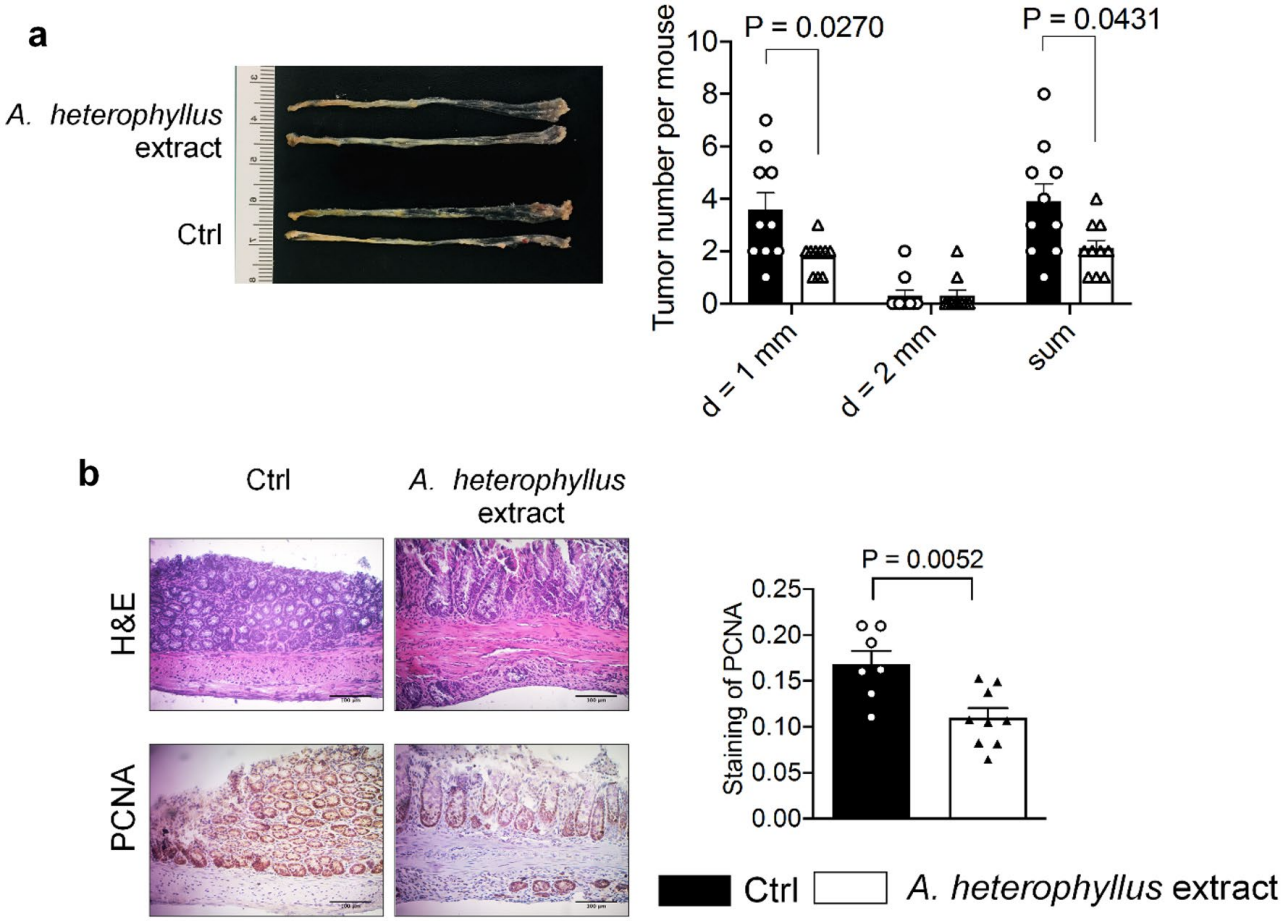
The *Artocarpus heterophyllus* extract suppresses AOM/DSS-induced colon tumorigenesis in mice. (**a**) Quantification of colon tumorigenesis in mice. Dissected colons were examined for number and dimension of tumors in each group and categorized on the basis of diameter (approximately 1 mm, 2 mm, and total sum). (**b**) H&E staining and immunochemical staining of PCNA in the colon scale bar=100μm. The results are expressed as the mean ± SEM, *n* = 11 mice in Ctrl group and *n* = 10 mice in the *A. heterophyllus* group. Ctrl, vehicle control; d, diameter.

**Figure 6:**
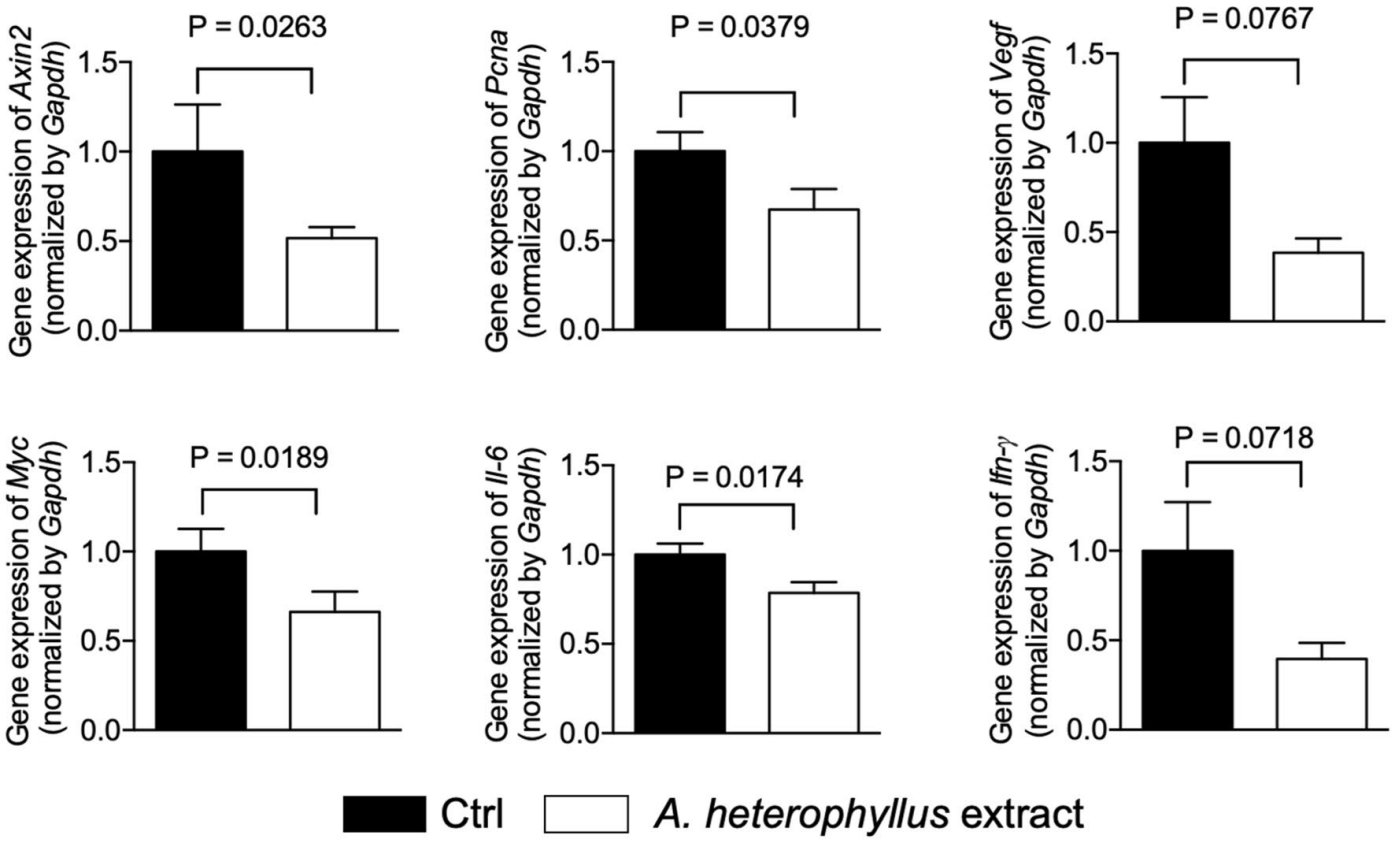
Expression of proinflammatory and protumorigenic genes in the colon after treatment with the *Artocarpus heterophyllus* extract. The results are expressed as the mean ± SEM, *n* = 11 mice in Ctrl group and *n* = 10 mice in the *A. heterophyllus* group. Ctrl, vehicle control.

Oral administration of 480 ppm *A. heterophyllus* extract suppressed AOM/DSS-induced colon tumorigenesis by 46.2%. Compared with vehicle-treated AOM/DSS mice, the *A. heterophyllus* extract-treated mice had a significantly *(p* = 0.0270) lower number of tumors per mouse, particularly those with a diameter of ~ 1 mm (Fig. 5a). The extract was well tolerated, with no fatalities observed within the test group. Immunohistochemical staining showed that treatment with the *A. heterophyllus* extract reduced the protein expression of tumorigenic marker proliferating cell nuclear antigen (PCNA) in the colon tissues (Fig. 5b). Furthermore, treatment with the *A. heterophyllus* extract significantly reduced the gene expression of a series of protumorigenic and proinflammatory genes (*Axin2* (*p* = 0.0379), *Pcna* (*p* = 0.0263)*, Il-6* (*p* = 0.0174)*,* and *Myc* (*p* = 0.0189)), while a substantial (*albeit* nonsignificant) decrease in the expression of *Ifn-γ* (*p* = 0.0718) and *Vegf* (*p* = 0.0767) was seen (Fig. 6). Overall, these results demonstrate the anti-CRC effects of the *A. heterophyllus* extract.

## Discussion

Using numerous in vitro and in vivo tools, we herein demonstrate the promising application of a prepared botanical wood extract (*A. heterophyllus* extract, 84% (*w*/*v*) artocarpin) in the reduction of tumor multiplicity, proinflammatory biomarkers, and gene expression and activities of cytochrome P450 2C enzymes, indicative of potential chemoprevention.

The extract was well tolerated in mice with 100% survival for the duration of this study, similar to previous studies involving the active component artocarpin, which suggests an acceptable safety profile^18^. Furthermore, cytotoxicity to cancer cells was observed using HCT116 colorectal cells with IC_50_ values < 10 mg/L (6.48 mg/L), along with less than half such impact on normal colon cell line CCD-18Co with an IC50 value of 17.8 mg/L, over a 48 h period. These values compared well with previous estimations of IC_50_ for purified artocarpin treated-colorectal cell lines SW480, HT-29, HCT15, and HCT116 with IC_50_ values of ~ 15 µM (or 6.55 mg/L) at 48 h, along with a nominal impact on the normal colon cell line CCD-18Co^18^. A time- and concentration-dependent effect on HCT116 cells was illustrated by the drop in IC_50_ after 48 h (6.48 ± 0.63 mg/L) and 72 h (4.23 ± 0.08 mg/L) in comparison to the value at 24 h (9.38 ± 1.26 mg/L), suggesting the likely exploitation of a pathway impacting cellular replication by the extract or its metabolites. Previously, artocarpin was proven to be cytotoxic toward a wide range of other cancer cells lines, including breast (T47D and MCF-7), bone (U-2 OS, MG63, and HOS), colon (SW480, HT-29, and HCT15), lung (A549), prostate (PC-3), and the CNS (U-87, MGU87, and U118)^18,22–26^, in addition to in vivo efficacy in breast, bone, colon, and prostate models^18,22,26–28^.

Recently, Wang and colleagues^13^ found that the expression of CYP2C9 and CYP2C19 (both mRNA and protein) was elevated in human colon cancer HCT116 and Caco-2 cells in comparison to the normal cell line CCD-18Co; they also demonstrated that targeted inhibitors could aid in mitigating tumorogenesis in mice. Our current results provide evidence that the presence of the *A. heterophyllus* extract could mitigate the in vivo gene expression of *Cyp2c37* and reduce the in vitro activities of human recombinant CYP2C9 and 2C19 isoforms, thereby providing strong evidence for its inhibitory and antagonistic effect on CYP2C9’s role in CRC. Modulation of CYP gene expression, as well as the direct binding to and inhibition of overexpressed enzymes in cancer, could lead to mitigation of carcinogenesis and tumor progression^29^. We also found that the extract behaves as an irreversible inhibitor (Fig. 3), providing it with the potential advantage of a lasting impact. Future studies considering chronic exposure and its impact on co-administered drugs reliant on CYP2C9 metabolism^9^ would allow expanding its safety profile.

Following the promising in vitro results, we embarked on a study of the impact of the *A. heterophyllus* extract in the AOM/DSS mouse model. The results obtained herein show that oral administration of the *A. heterophyllus* extract, as the component of a diet fed to AOM/DSS-induced colon tumorigenesis mice, reduced tumor multiplicity, consistent with the findings of a previous study utilizing a pure artocarpin gavage^18^. Moreover, according to Fig. 6, the expression of proinflammatory and protumorigenic genes (*Axin2*, *Ifn-γ*, and *C-myc*) was downregulated, partially supporting a previous murine study which showed that topical application of artocarpin resulted in the decreased protein expression of proinflammatory cytokines IL-1β and TNF-α^30^. While there was a decrease in the genetic expression of *Il-1β* and *Tnf-α* in this study, it was not significant (data not shown).

Previous research has suggested the arachidonic acid/COX-2 pathway via CYP activation as a key player in the inflammatory response driving colitis-associated colon tumorigenesis^13,30^. Furthermore, a previous in vitro experiment determined that artocarpin, like celecoxib, acts as a COX-2 inhibitor with anti-inflammatory properties, which, in the presence of AOM/DSS, results in an increase in inflammatory response^18,31^. DSS-induced colitis, caused by the microrupture of colon epithelial tissue, is characterized by the infiltration of immune cells as an inflammatory response^32,33^. We identified a decrease in the gene expression of proinflammatory cytokine *Il-6* and chemokine *Ifn-γ* (Fig. 6) in mice fed the *A. heterophyllus* extract. Artocarpin, as one of numerous *Artocarpus* spp.-derived phytochemicals, has been shown in numerous studies to behave as an anti-inflammatory and anticancer agent, which suggests a potential role in addressing the inflammation seen in colitis-associated cancer^27,34^. These results demonstrate that, while the extract could repress the proinflammatory response, its antitumor effects, similar to celecoxib, may not depend solely on its anti-inflammatory properties^31,35^.

There was a significant reduction in tumor proliferation, as well as a reduction in crypt cells marked by PCNA staining. Additionally, H&E staining of colon tissue showed greater inflammation in control mice, suggesting that treatment with the *A. heterophyllus* extract aids in the recovery from AOM/DSS-associated colitis tumorigenesis.

In summary, the administration of an extract from *A. heterophyllus* Lam., standardized to contain 84% (*w*/*v*) artocarpin, effectively suppressed tumor multiplicity and inflammation in AOM/DSS-induced mouse colon via decreasing the expression of cytokine *Il-6*. Additionally, the reduced expression of the *Cyp2c37* gene in mice and the potent inhibition toward the activity of human CYP2C9, an upregulated isoform in colon cancer cells, point to the chemopreventive potential of this extract. Taken together, the *A. heterophyllus* extract with its active component, artocarpin, displays promising anti-inflammatory, anticancer, and chemopreventive properties and may be a lead molecule for treating colitis-associated tumorigenesis.

## Materials and methods

### Preparation of crude polar and nonpolar plant extracts

*Artocarpus heterophyllus* Lam. (Voucher # 36378), collected at the Botany Gardens, the University of the West Indies, Mona-Campus, Jamaica, was authenticated by Mr Patrick Lewis, the local herbarium curator. Oven-dried wood chips (1200 g), fruit rind chips (323 g), ground testa (24 g), and seeds (170 g) were exhaustively extracted with *n-*hexane and then methanol, at least 3 times. Crude extracts were concentrated to dryness *in vacuo* on a rotary evaporator (Heidolph Laborota 4000 efficient and BUCHI water bath B-481) below 55 °C, and they were stored at − 20 °C in aluminum-wrapped glass vials until reconstituted in appropriate solvents for cytotoxicity evaluation.

### Semi-purification of crude methanol extract via silica gel column chromatography

Following the determination of optimum cytotoxicity, the methanol crude extract of the wood chips (red–orange gum, 3 g) was subjected to further separation using silica gel 60 Å (230–400 mesh (40–63 μm) particle size, SiliCycle Inc., Quebec City, QC, Canada) gravity column chromatography. The crude methanol wood extract was added to the column (60 × 6 cm) and eluted with 90% *n*-hexane with increasing concentrations of ethyl acetate. This was followed by neat ethyl acetate, which was gradually enriched with methanol to a maximum concentration of 75% (*v*/*v*), to produce 14 different semi-purified fractions (I–XIV). These were monitored using TLC via molybdenum spray detection and combined according to Rf values. The fractions were concentrated, and the resulting powdered extracts were stored as previously described, before subsequent use in cytotoxicity evaluation against colorectal (HT29), prostate (PC3), and liver (HepG2) cancer cell lines (Table S1), subject to the quantity of material available.

### Preparation and standardization of an enriched *A. heterophyllus* extract

Guided by the conditions that yielded the most potent fractions from chromatographic separation (above), an enriched extract was prepared, as briefly described elsewhere^36^. Oven-dried wood chips (2.5 kg) of *A. heterophyllus* Lam. were exhaustively defatted with *n-*hexane and then differentially re-extracted in methanol in amber containers. The methanol crude extract (21.2 g, yield: 0.85%), concentrated as previously described, was suspended in water and subjected to successive portioning with equivalent volumes of *n-*hexane followed by methylene chloride. The methylene chloride portion was subsequently recrystallized with ethyl acetate/hexane, producing a yellow-orange powder (referred to as the *A. heterophyllus* extract, 4.5 g, yield: 0.18%), standardized for artocarpin (Fig. 1) and stored at − 20 °C in an aluminum-wrapped glass vial until further use.

To quantitatively determine artocarpin content in the extract, it was dissolved to a concentration of 1.53 mg/mL in methanol, before injecting 2 µL into a quantitative high-performance liquid chromatograph (HPLC) mass spectrometer (MS) equipped with UV diode array detection (DAD) (Velos-Pro, Thermo Fisher Scientific). A Phenomenex C18 column (150 × 3 mm, 3 μm particle size) was used as the stationary phase, and the mobile phase at a flow rate of 0.4 mL/min was applied as follows: 0–1 min = isocratic gradient 10% acetonitrile in H_2_O; 1–20 min = linear gradient 10% acetonitrile in H_2_O to 10% acetonitrile in methanol. Pure artocarpin (BOC Sciences NY, USA) was used to produce a calibration curve at concentrations of 1.1 mg/mL, 0.55 mg/mL, and 0.257 mg/mL, using 2 µL injections. Concentrations were calculated from the integrated UV peak areas at a wavelength of 256 nm. HPLC-grade solvents were used.

### In vitro CYP P450 enzyme assays

High-throughput fluorometric inhibition assays performed in 96-well microtiter plates were used to evaluate the inhibitory capacity of the *A. heterophyllus* extract on human CYP2C enzyme activity. Catalytic activities describing the CYP2C9-mediated conversion of 7-methoxy-4-trifluoromethylcoumarin (MFC) demethylase (80 µM) to fluorescent 7-hydroxy-4-trifluoromethyl-coumarin (HFC) and 3-cyano-7-ethoxycoumarin (CEC) deethylase (25 µM) to fluorescent 3-cyano- 7-hydroxycoumarin (CHC)^37,38^ were monitored. For inhibition assays, the incubating mixture contained CYP2C9 (Cypex Ltd.) (2.5 pmol) and CYP2C19 (Cypex Ltd.) (1.0 pmol), in 0.1 M potassium phosphate buffer (KPB, pH 7.4) in a final volume of 0.2 mL, which was prewarmed for 10 min, unless otherwise stated^37^. Following the appropriate incubation period (CYP2C9, 45 min and CYP2C19, 30 min), 75 μL of 80% acetonitrile/20% Tris base (0.5 M) was added to stop the reaction. Fluorescence generated from the formation of CHC or HFC was recorded using a Varian Cary Eclipse Fluorescence Spectrophotometer microplate reader with excitation/emission wavelengths of 409/460 nm and 405/530 nm, respectively. Methanol (1.5% *v*/*v*) was used as a negative control.

### ***Direct inhibition studies (IC***_***50***_*** determination)***

The prewarmed *A. heterophyllus* extract was serially diluted (23.27–0.01 µg/mL) in tubes containing human CYP2C enzymes and their respective substrates, at concentrations equal to their K_m_ values^37^. NADPH (43 µM) was added to initiate the reaction. After terminating the reaction, fluorescence data were measured and converted to CYP activity ± SEM as a percentage of the control using Microsoft Excel (2002). IC_50_ values were determined by fitting the average % CYP activity ± SEM with a four-parameter logistic (4PL) nonlinear regression model, using SigmaPlot 10.0 from Systat Software Inc., San Jose California USA, www.systatsoftware.com.

$$\%\,\mathrm{Enzyme}\,\mathrm{activity}=\mathrm{ Min }+\frac{\left(\mathrm{Max }-\mathrm{ Min}\right)}{\left(1+{10}^{\left(\left(\mathrm{Log IC}50 -\mathrm{ x}\right) \times \mathrm{ Hill slope}\right)}\right)},$$

where Min and Max are the minimal and maximal observed effects, respectively, x is the concentration of the test agent, and IC_50_ is the half-maximal inhibitory concentration.

In order to determine the kinetics of the *A. heterophyllus* extract on CYP2C9 activity, dialysis, and IC_50_ shift assays were carried out. To evaluate reversibility, CYP2C9 was preincubated with an IC_75_ concentration of extract in a Pur-A-Lyzer™ Midi 60,010 dialysis unit (Sigma Aldrich) or microcentrifuge tube for 8 h at 4 °C. The residual enzyme activity of the incubation mixtures pre and post dialysis was expressed as a percentage of their respective vehicle controls (0.18% methanol). For the IC_50_ shift assay, the *A. heterophyllus* extract in the presence of CYP2C9 (5 pmol) was directly incubated with substrate (160 µM) or first preincubated with or without NADPH for 30 min.

### Cell lines and culture

The human colon adenocarcinoma cell line HCT116 (ATCC, CCL-247) and normal cell line CCD-18Co (ATCC, CRL-1459) were cultured in DMEM, supplemented with 10% fetal bovine serum, and maintained under standard cell culture conditions at 37 °C and 5% CO_2_ in a humidified incubator^39^. To rule out specificity to one type of colon cancer cell, the aggressive HCT116 cell line was used, as HT29 cells were employed earlier in the study (Table S1).

### Cell viability assay

Viable cells were quantified using a tetrazolium-based colorimetric 3‐(4,5‐dimethylthiazol‐2‐yl)‐2,5‐diphenyl‐2*H*‐tetrazolium bromide (MTT) assay, by determining the amount of formazan crystals produced by metabolically active cells^40,41^. Following 24 h of incubation, cells seeded into 96-well plates (1–1.5 × 10^3^ cells per well) were exposed to the *A. heterophyllus* extract or ethanol (3% *v*/*v*) for a further 24, 48, or 72 h. After disposal of used medium, MTT in fresh medium (0.5 mg/mL) was added to each well. The plate was incubated for 1–2 h, after which the medium was discarded and the formed formazan crystals were dissolved in DMSO (100 µL). The absorbance of cells was measured at 570 nm using a BioTek Synergy 2 microplate spectrophotometer with Gen5 Software (Bio-Tek). The data are presented as the percentage of viable cells using SigmaPlot 10.0, and the IC_50_ values were determined.

### Animal experiments

The mouse experiments were conducted in accordance with the protocols approved by the Institutional Animal Care and Use Committee of the University of Massachusetts Amherst (protocol number: 2017–0019) with mice maintained in a specific pathogen-free (SPF) facility of the University of Massachusetts (Amherst, MA). C57BL/6 male mice (age: 6 weeks) were purchased from Charles River (Cambridge, MA). All methods involving animals are reported in accordance with ARRIVE guidelines^42^.Upon arrival, the mice were allowed free access to water and a chow diet for 1 week acclimation. Afterward, mice were treated with 10 mg/kg AOM (Sigma Aldrich) via intraperitoneal injection, and at week 1 post AOM injection, they were given 2% DSS (36–50 kDa, MP Biochemicals) in drinking water for 1 week. Mice were then randomly assigned to two groups (n = 11 in control group, (mean ± SEM) body weight = 23.02 ± 0.39 g; and n = 10 in *A. heterophyllus* group, (mean ± SEM) body weight = 22.94 ± 0.46 g) and maintained ad libitum on a modified AIN-9G diet (containing 10% fat). Corn oil was the only source of fat content, and the diet composition, as described in Supplementary Table S2, contained 480 mg of *A. heterophyllus* extract/kg diet dissolved in polyethylene glycol 400 (PEG 400, Millipore) as a vehicle (0.1% in diet, *v*/*w*) or vehicle alone for 6 weeks. All ingredients except the corn oil and *A. heterophyllus* extract were purchased from Dyets Inc. Bethlehem, PA. The diets were freshly prepared and changed twice weekly. At week 8 post AOM injection, the mice were sacrificed, and colon tissues were collected for analysis. Colon tissues were cut open longitudinally, washed in PBS, and inspected under a dissecting microscope. The size of the tumors was determined using the following formula: tumor size = π × d^2^/4, where d is the diameter of each tumor. Tissue staining and qRT-PCR analysis of gene expression in colon tissues were carried out according to protocols described in the Supplementary Materials and Methods using the RT-PCR primers listed in Supplementary Table S3.

### Statistical analysis

Data are expressed as the mean ± SEM. Significance comparisons for in vitro data were performed using a two-tailed Student *t*-test with data analysis performed using SigmaPlot 10.0, while comparisons between two groups (in vivo) were performed using the Mann–Whitney test with data analysis performed using Prism 6 (GraphPad Software). A *p* value < 0.05 was considered as statistically significant.

## Supplementary Information

Supplementary Tables.
