# Supplementary material for: Potential chemopreventive, anticancer and anti-inflammatory properties of a refined artocarpin-rich wood extract of Artocarpus heterophyllus Lam
Source: Sci Rep. 2021 Mar 25;11:6854. doi: 10.1038/s41598-021-86040-5 (PMC7994669; doi:10.1038/s41598-021-86040-5)
Supplement: Supplementary file 1 — Supplementary Tables. [file 41598_2021_86040_MOESM1_ESM.docx]

**Potential chemopreventive, anti-cancer and anti-inflammatory properties of a refined artocarpin-rich wood extract of *Artocarpus heterophyllus* Lam.**

Isaac J. Morrison^1,2^**^ǂ^**, Jianan Zhang^3^**^ǂ^** , Jingwen Lin^3^, JeAnn E. Murray^1^, Roy Porter^4^, Moses K. Langat^5^, Nicholas J. Sadgrove^5^, James Barker^6^, Guodong Zhang^3^ and Rupika Delgoda^1^**^*^**

**Supplementary Data**

**Table S1**. **Preliminary bioactivity screening for semi-purified extracts of *A. heterophyllus* Lam. against prostate (PC3), colon (HT29) and Liver (HepG2) cancer cell lines for 24 h via MTS assay.**

| **Semi-purified**  ***A heterophyllus fractions*** | **IC_50_ (μg/mL) with cell lines** | | |
| --- | --- | --- | --- |
|  | **HT29** | **PC3** | **HepG2** |
| I | >375^a^ | >375^a^ | ND |
| II | >312 ^a^ | >234 ^a^ | >312 ^a^ |
| III | >412 ^a^ | >354 ^a^ | <88^b^ |
| IV | >518 ^a^ | >388.5 ^a^ | >120 ^a^ |
| V | 67.1±0.9 | 69.2±3.5 | <32.5^b^ |
| VI | 22.7±0.6 | <30^b^ | 16.2±0.5 |
| VII | 30.1±0.1 | 26.0±0.9 | 16.5±0.8 |
| VIII | 16.3±0.9 | 24.0±0.9 | <25.4 ^b^ |
| IX | <60 ^b^ | <60 ^b^ | 40.3±5.1 |
| X | 22.9±1.1 | 50.4±3.2 | 32.0±5.3 |
| XI | 41.1±0.5 | 50.5±1.7 | 76.8±10.7 |
| XII | >188 ^a^ | >188 ^a^ | ND |
| XIII | >180 ^a^ | >180 ^a^ | ND |
| XIV | >951^a^ | 748.5±29.4^a^ | ND |

Table displays assessments of biological activity of semi-purified fractions emanating from silica gel column, from the the crude methanol wood extract. The cell viability (in triplicates) was assessed using the MTS assay after a 24h exposure to colon (HT29), prostate (PC3) and liver (HepG2) cancer cell lines, in that order. Briefly, cell lines obtained from ATCC were cultured in McCoy’s 5A, F12K and EMEM media supplemented with 10% fetal bovine serum and penicillin (100 U/mL)/streptomycin (100 g/mL) respectively under standard cell culture conditions at 37°C and 5% CO_2_ in a humidified incubator^39^.

^a^ values represent the effect of the maximum concentration of extract tested. ^b^ values represent concentration below which the IC_50_ value lies. ND-Not Done, due to insufficiency of material emanating from the column. Fractions V-XI were noted as the most active.

**Table S2. Composition of the experimental diets to study the effects of *A. heterophyllus* extract on colorectal cancer**. In the table provided the quantity of ingredients that formed a part of the diet for the mice was provided along with the added extract for the experimental group.

| **^a^Ingredient (g/kg)** | **Control diet** | ***A. heterophyllus* extract diet** |
| --- | --- | --- |
|  |  |  |
| Casein | 200 | 200 |
| L-Cystine | 3 | 3 |
| Sucrose | 100 | 100 |
| Dyetrose | 132 | 132 |
| Cornstarch | 367.5 | 367.5 |
| Cellulose | 50 | 50 |
| Mineral Mix #210025 | 35 | 35 |
| Vitamin Mix #310025 | 10 | 10 |
| Choline Bitartrate | 2.5 | 2.5 |
| Corn oil | 100 | 100 |
| *A. heterophyllus* extract  (dissolved in PEG400) | 0 | 0.48 |

^a^All ingredients except the corn oil and *A. heterophyllus* extract were purchased from Dyets Inc. Bethlehem, PA

**Table S3**. **Sequences of mouse primers in qRT-PCR**. In this table the list of sequences for the various mouse primers used is provided.

| **Gene** | **Forward primer** | **Reverse primer** |
| --- | --- | --- |
| Mouse *Gapdh* | AGGTCGGTGTGAACGGATTTG | TGTAGACCATGTAGTTGAGGTCA |
| Mouse *Il-6* | TAGTCCTTCCTACCCCAATTTCC | TTGGTCCTTAGCCACTCCTTC |
| Mouse *Ifn-γ* | ATGAACGCTACACACTGCATC | CCATCCTTTTGCCAGTTCCTC |
| Mouse *Axin 2* | TGACTCTCCTTCCAGATCCCA | AGCCCACACTAGGCTGACA |
| Mouse *Myc* | ATGCCCCTCAACGTGAACTTC | GTCGCAGATGAAATAGGGCTG |
| Mouse *Vegf* | GCACATAGAGAGAATGAGCTTCC | CTCCGCTCTGAACAAGGCT |
| Mouse *Pcna* | TTTGAGGCACGCCTGATCC | GGAGACGTGAGACGAGTCCAT |
| Mouse *Cyp2c37* | AATGGAATGGGCCTTGCA | GCAACGTGCTTCTTCTTGAACG |
